# Preoperative Jugulofemoral Shunting in Complex Extended Thymectomy—Anesthetic Optimization for Invasive Thymoma

**DOI:** 10.1016/j.atssr.2026.02.004

**Published:** 2026-02-19

**Authors:** Tiffany To, Hoi Leong Chum, Ki Kwong Li, Ho Yan Howard Chan, Wai Hon Chu

**Affiliations:** 1 School of Medicine, University of Tasmania, Hobart, Tasmania, Australia; 2 Department of Cardiothoracic Surgery, Queen Elizabeth Hospital, Hong Kong; 3 Department of Anaesthesiology, Queen Elizabeth Hospital, Hong Kong

## Abstract

Malignant thymomas infiltrate adjacent blood vessels and mediastinal structures, posing challenges to surgical treatment. A 50-year-old man, who was diagnosed with type AB thymoma (Masaoka stage IVa) adhering to both superior vena cava (SVC) and bilateral innominate veins, underwent an extended total thymectomy with vascular reconstruction through a median sternotomy. Given the invasive nature of the large thymoma, which required SVC clamping and repair, a jugulofemoral shunt was introduced to improve hemostasis and minimize the risk of cerebral venous hypertension intraoperatively. This case highlights the successful application of preoperative venovenous shunting, emphasizing its role in preventing complications associated with SVC clamping.

Thymoma, which is a common primary tumor in the anterior mediastinum, is a cancer of the thymus gland.^1^ Despite recent development of minimally invasive techniques for thymectomy, such as video-assisted thoracoscopic surgery, subxiphoid single-port, and robotic approach, median sternotomy remains the gold standard to manage locally invasive thymomas.^2^ This report presents a case of a large thymoma invading the superior vena cava (SVC) and bilateral innominate veins in a 50-year-old man. An extended total thymectomy and vascular reconstruction were performed with additional techniques for anesthetic optimization. This aims to emphasize the importance and viability of jugulofemoral shunting in enhancing perioperative management.

A 50-year-old man was referred to the cardiothoracic surgery clinic with incidental findings of an anterior mediastinal mass on chest roentgenogram. He was asymptomatic at presentation, with no significant medical history or autoimmune disorders. He was a chronic smoker, with 2 packs of cigarettes per day, and a social drinker.

Chest computed tomography (CT) showed a right-sided anterior mediastinal mass invading the superior vena cava (SVC) as well as the innominate veins (Figure 1). Imaging with 18-fluorodeoxyglucose positron emission tomography combined with CT also revealed an anterior mediastinal mass (12.6 × 8.5 × 4.3 cm), showing an increased 18-fluorodeoxyglucose uptake, with a maximum standardized uptake value (SUVmax) of 8.5. An 8-mm right pleural nodule, suspicious of metastasis, was identified with an SUVmax of 2.8, and a 1.1-cm right hilar lymph node was noted with an SUVmax of 2.1. The mass was then confirmed to be a thymoma through CT-guided fine-needle aspiration cytology. Therefore, an extended total thymectomy through a median sternotomy was performed.

**Figure 1:**
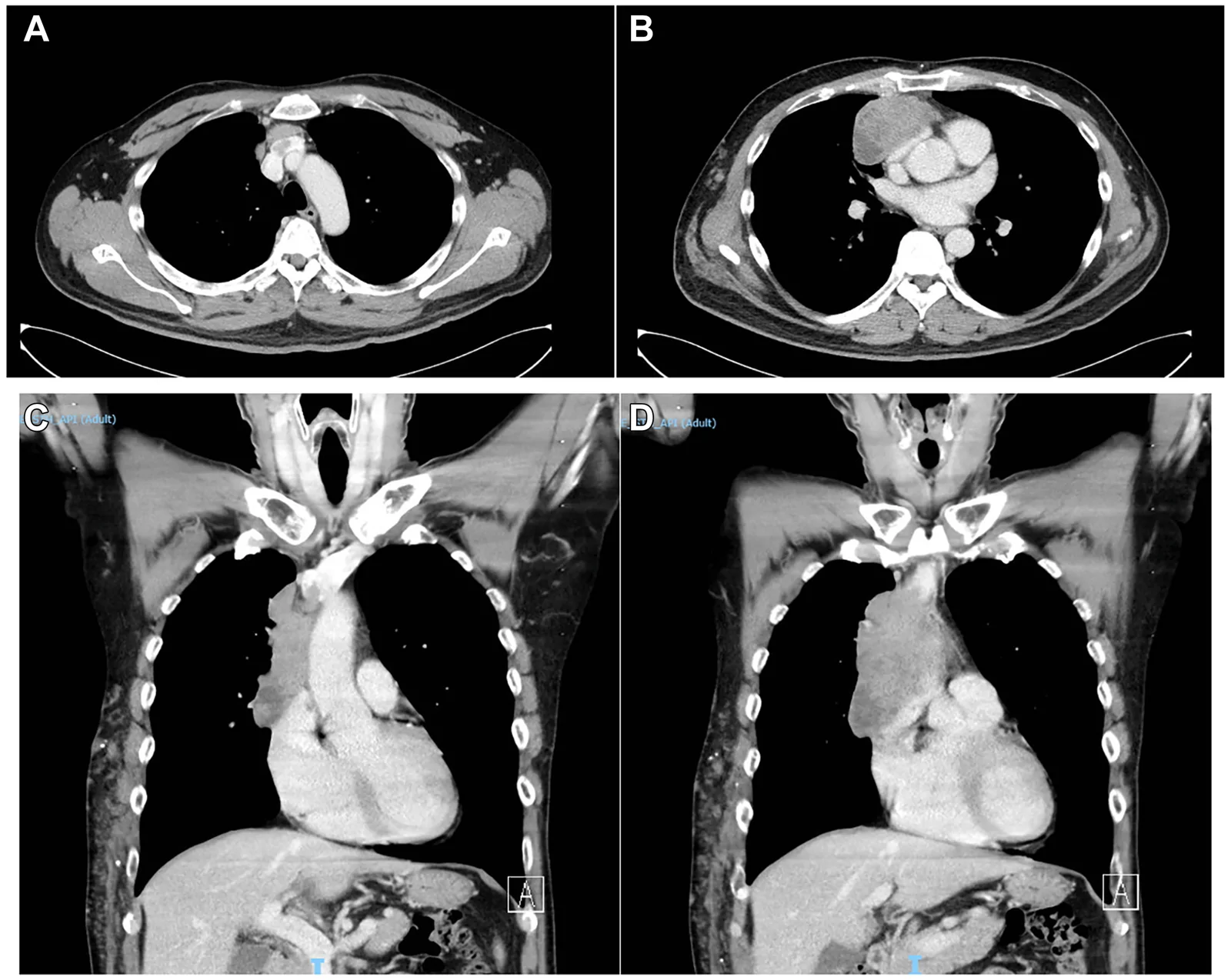
Computed tomography shows a right-sided anterior mediastinal mass invading superior vena cava and innominate veins. (A, B) Transverse views. (C, D) Coronal views.

Because SVC clamping and repair were required due to the invasive nature of the thymoma, preoperative jugulofemoral shunting was conducted to optimize hemostasis and lower the risk of cerebral venous hypertension. Two 8.5F Swan-Ganz sheath catheters were inserted to the right and left jugular veins with pressure monitoring through the side port, respectively. A 12F triple-lumen dialysis catheter was also introduced to the right femoral vein, serving as the return catheter from the Swan-Ganz catheters (Figure 2). Thus, a temporary SVC bypass was established for intraoperative use.

**Figure 2:**
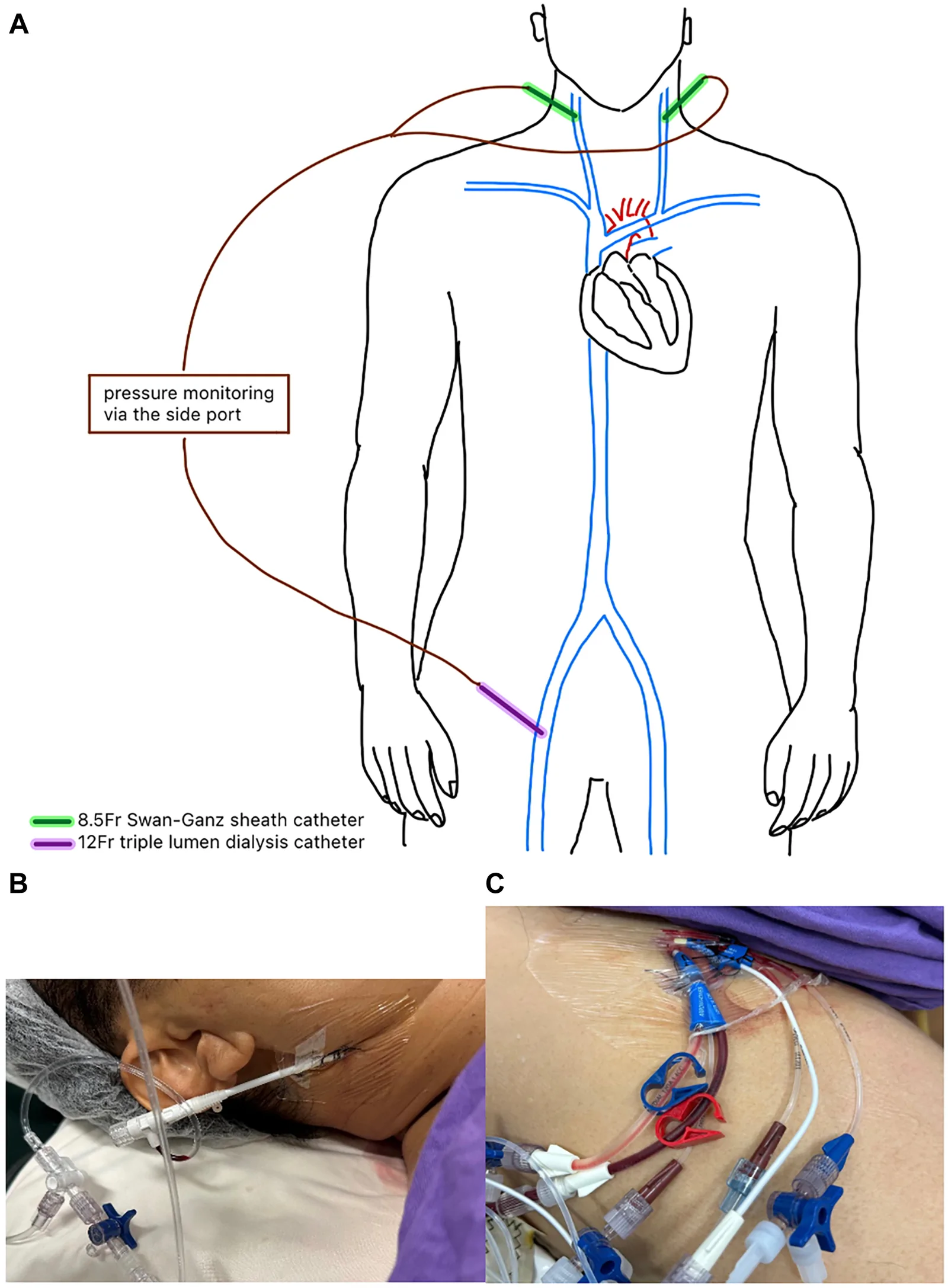
Jugulofemoral venovenous shunt. (A) Schematic illustration of the shunt configuration. (B) An 8.5F Swan-Ganz sheath catheter was inserted to right jugular vein. (C) A 12F triple-lumen dialysis catheter was introduced to right femoral vein.

Median sternotomy was performed, and the pericardium was opened. The tumor was located near the confluence of SVC and bilateral innominate veins. Further dissection revealed partial obstruction of the proximal left innominate vein as well as partial invasion into the walls of both the SVC and right innominate vein. The tumor was also densely adhered to the pericardium over the SVC and aorta, along with the right upper lobe of the lung, with multiple fleshy projections extending into the right pleural cavity.

To facilitate tumor resection and vascular reconstruction, the left innominate vein was first exposed and isolated, with its distal end clamped. The right atrial appendage was clamped and opened, followed by the creation of a 16-mm ringed polytetrafluoroethylene graft anastomosis between the right atrial appendage and left innominate vein. The right innominate vein and SVC were then isolated and clamped. Jugulofemoral shunting, which was prepared preoperatively, was initiated to establish a temporary SVC bypass.

The thymus was resected with the involvement of vessel junction between SVC and right innominate vein, which was later repaired by a pericardial patch. The tumor, adhered to the right upper lobe of the lung, was dissected free through a wedge resection using an ECHELON+ stapler (Ethicon). A biopsy sample of the right pleural nodule, suspicious of metastasis, was also taken.

Pathology reports later revealed the thymectomy specimen and the pleural nodule were both consistent with type AB thymoma (Masaoka stage IVa). The surgery was unremarkable, and the patient recovered well postoperatively with no complications. He was then referred to oncology for ongoing monitoring and surveillance CT.

## Comment

Locally invasive thymoma is difficult to manage due to its proximity to nearby vasculatures, making surgical resection more complicated. With continuous surgical and technological advancement, extended thymectomy has become less controversial and is also associated with better long-term outcomes.^3^ When the tumor is invading the SVC and innominate veins, complex vascular procedures, such as clamping, repair, and reconstruction of blood vessels, become necessary. These significantly increase the perioperative risks, requiring meticulous monitoring to prevent hemodynamic instability, cerebral venous hypertension, and neurologic complications.^4^

Apart from pharmacologic blood pressure support, cardiopulmonary bypass, and temporary innominate-to-right-atrial bypass grafting, the technique of jugulofemoral shunting was introduced by Perentes and colleagues.^5^ However, this preoperative strategy has not been adequately explored in the current literature, leading to limited application around the globe.

SVC clamping is common in operations involving the resection of lung cancers and mediastinal malignancies, including invasive thymoma.^6^ To minimize the risks of this procedure, venovenous shunting is found to be a safe and simple alternative to maintain hemostasis perioperatively.^7^ There are 2 major types of venovenous shunting, namely, internal and external. An internal venovenous shunt, which is established intraoperatively, connects the innominate vein to the right atrium, whereas a preoperative external venovenous shunt involves the internal jugular vein and femoral vein. Jugulofemoral shunting, used in this case, is favored for its simplicity because only Swan-Ganz sheath catheters are required, whereas internal venovenous shunting requires specialized cardiac expertise and occupies the surgical field.

During the operation, clamping of the SVC creates a gradient between the jugular and femoral veins, driving blood to flow along the shunt without any pumping devices. The shunt length is also minimized, and prophylactic heparin is used to mitigate the risk of tube thrombosis.^5^ Nevertheless, the shunt retains inherent limitations, and minimizing SVC clamping time remains the priority.

Given the substantial evidence supporting the safety and efficacy of preoperative jugulofemoral shunting, we recommend the routine application of external venovenous shunting in cases involving potential SVC clamping and reconstruction, which is aligned with recent literature.^8^ Increased awareness and use of this technique may enhance perioperative outcomes by improving hemodynamic stability and preventing cerebral venous hypertension. Future research is encouraged to investigate its broader applicability and long-term benefits in the resections of mediastinal tumors.

This case of successful extended total thymectomy with vascular reconstruction and optimized anesthetic strategies highlights the importance and viability of preoperative jugulofemoral shunting, which aims to improve venous return, minimize the risk of cerebral venous hypertension, and enhance perioperative management. Its simplicity and effectiveness make it a valuable technique in the management of locally invasive thymoma as well as complicated surgeries involving SVC clamping.
